# Ginkgolide B improved postoperative cognitive dysfunction by inhibiting microgliosis-mediated neuroinflammation in the hippocampus of mice

**DOI:** 10.1186/s12871-022-01750-1

**Published:** 2022-07-18

**Authors:** Ting Luo, Ya-Nan Hao, Dan-Dan Lin, Xiao Huang, An-Shi Wu

**Affiliations:** Department of Anesthesiology, Beijing Chao-Yang Hospital, Capital Medical University, No. 8, Gongtinan Road, Chaoyang District, Beijing, 100020 China

**Keywords:** Ginkgolide B, Platelet activating factor, Postoperative cognitive dysfunction, Microglia, Neuroinflammation, Surgery

## Abstract

**Background:**

Postoperative cognitive dysfunction (POCD) are a common complication of the central nervous system following surgery and anesthesia. The specific pathogenesis and effective therapeutics of POCD need to be further studied. Ginkgolide B (GB), a platelet-activating factor receptor-specific antagonist, has been suggested to have strong anti-inflammatory effects. Here we tested the effects and mechanism of GB on POCD of aged rats.

**Methods:**

Neurobehavioral tests were used to investigate the effect of GB pretreatment on POCD. The hippocampus were harvested to test the expression of proinflammatory cytokines by ELISA. The expression of the microglial marker ionized calcium-binding adaptor molecule-1 (Iba-1) in the hippocampus was evaluated by western blot assay and immunohistochemistry. A Nissl staining experiment was used to detect the neuronal numbers in the hippocampus.

**Results:**

Surgery might result in the overexpression of platelet activating factor (PAF) in the plasma and hippocampus and might cause hippocampus-dependent memory impairment. GB pretreatment, inhibited the activation of microglia, reduced the levels of IL-1β and TNF-α, decreased the loss of neurons after surgery, and prevented POCD in aged rats.

**Conclusion:**

Our findings suggested that PAF was involved in the development of POCD. Improvement of POCD by PAF antagonist GB was associated with the inhibition of microgliosis-mediated neuroinflammation and neuronal apoptosis in aged rats.

**Supplementary Information:**

The online version contains supplementary material available at 10.1186/s12871-022-01750-1.

## Background

Postoperative cognitive dysfunction (POCD) [1] are a common complication of the central nervous system in postoperative period. POCD are characterized by impairments in memory, concentration, language comprehension, abstract thinking and social integration. It has been reported that POCD often occurs after surgery and can last for days to weeks. In some patients, POCD even become a permanent disorder. POCD have been shown to be associated with decreased quality of life and increased morbidity and mortality [2]. Thus, much attention has been given to the control of POCD. Although several pathophysiological mechanisms underlying POCD have been proposed, there are currently few effective interventions to prevent this disease.

Neuroinflammation has been suggested to play an important role in the pathogenesis of POCD [3]. Accumulating evidences have shown that increased production of proinflammatory cytokines, including interleukin-1 beta (IL-lβ), interleukin-6 (IL-6), and tumor necrosis factor alpha (TNF-α), are associated with POCD [4–6]. Inhibition of central proinflammatory cytokine signaling may attenuate postoperative memory impairment and other degenerative nervous system diseases [7–9]. Microglia are a type of primary immune cell in the central nervous system. Currently, the activation of microglia has been suggested to contribute to POCD by releasing proinflammatory cytokines [10, 11]. Hence, microgliosis mediated neuroinflammation may be a target for the treatment of POCD.

Platelet-activating factor (PAF), which is an endogenously synthesized phospholipid substance, is a potent proinflammatory mediator [12]. Recently, PAF has been implicated in neural injury, such as cognitive deficits, HIV associated dementia, ischemia–reperfusion injury [13–16]. Ginkgolide B (GB), which is a terpene lactone extracted from the *Ginkgo biloba* plant, is a kind of PAF receptor antagonist. GB plays a neuroprotective role in various brain diseases by inhibiting inflammation and nerve apoptosis [17–20].

Therefore, the present study aimed to investigate the effect of the PAF receptor antagonist GB on POCD and to determine whether GB exerts neuroprotection by suppressing microglial activation and neuroinflammation.

## Methods

### Animal selection and grouping

Thirty aged *Sprague–Dawley* rats (21 ± 1 months old, Chengdu Dossy Experimental Animals Corporation, Sichuan, China) weighing approximately 760 g ± 50 g were used in the studies. All the protocols were approved by the Animal Experiments and Experimental Animal Welfare Committee of Capital Medical University (ethical review number: 2018–0003; Beijing, China). The study was carried out in compliance with the ARRIVE guidelines. All the animals were treated according to internationally accepted principles. The rats were fed a standard diet and water in a controlled environment (the temperature was kept at 19–22℃, the humidity was kept at 40–60%), under a 12 h dark/12 h light cycle. These rats were randomly divided into three groups (*n* = 10) by randomized digital table as follows: (1) control group, which did not undergo any intervention; (2) surgery group, which underwent partial hepatectomy under isoflurane anesthesia; (3) GB group, which received GB (10 mg/kg, purity > 99%, supplied by National Institutes for Food and Drug Control of China) by intraperitoneal injection for 5 days before the partial hepatectomy. The aim of this study was not to explore the effect of anesthesia on POCD. Hence no anesthesia group was included in this study. And some previous studies about POCD didn’t include anesthesia group neither [21].

### Partial hepatectomy

Partial hepatectomy surgery was used to establish the model of POCD, following the methods described by Tian Y and Weri [22–24]. In brief, the rats were anesthetized with 2.1% isoflurane. After shaving the fur and sterilizing on the operating table, a 1.5 ~ 2-cm incision was cut along the midline of the abdomen to expose the liver. Then, we observed whether the anatomy of the liver was normal. We used 4–0 sutures at the root of the left hepatic lobe to ligate the hepatic duct and artery. When the color of the lobe became dark, we cut liver tissue along the knot. In addition, the rats were injected subcutaneously with saline for rehydration. The skin and muscle wounds were sutured layer by layer. Then, the rats were placed in a thermostatic container to maintain body temperature. When the rats awakened, they were returned to their individual cages. Blood oxygen saturation was monitored during surgery, and attention was paid to maintain the body temperature.

### Open field test

To assess the exploratory and locomotor activity of the rats, each rat was placed in the center of a black, opaque plastic chamber (60 × 60 × 30 cm) on the 3rd day after surgery. A video tracking system recorded each rat's locomotion activity for 5 min and analyzed the total distance traveled and the time spent in the center area of the chamber. Between the sessions, the chamber was cleaned with 75% ethyl alcohol.

### Trace fear conditioning test

The trace fear conditioning (TFC) test is a reliable method for testing hippocampal dependent memory in rodents. The day before the surgery, the rats were placed in a conditioning chamber (Xeye FCS, Tianming Hongyuan Technology Development Corporation, Beijing, China) and allowed to explore for 100 s. Then the rats were exposed to an auditory cue (75–80 Db, 5 kHz, conditional stimulus) for 30 s and then to a 2-s foot shock (0.8 mA; unconditional stimulus). The fear-conditioning paradigm was consisted of two trials of tone and foot-shock pairings. When the trial was finished, the rats returned to their cages. Twenty-four hours later, partial hepatectomy surgery was performed. On postoperative day 3, two hours after open field test, the rats were placed back into the TFC chamber but were not subjected to any tone or shock for 5 min for contextual testing (a hippocampus-dependent task). The movement of the rats was monitored by an attached tracking insight system. Two hours later, the rats were placed in a different chamber for the cued fear test (a hippocampus independent task). The rat was allowed to explore for 3 min. Then, the training tone was delivered for an additional 3 min, and the freezing behavior of the rats was recorded.

### Enzyme-linked immunosorbent assay

After the behavioral tests, the rats were anesthetized with pentobarbital solution (50 mg/kg, intraperitoneally). Blood was collected in the ethylenediaminetetraacetic (EDTA) coated tubes by cardiac puncture. And then the rats were sacrificed by decapitation. The hippocampal tissues were immediately harvested, dissected on ice, and then weighed. A certain amount of phosphate-buffered saline was slowly added, which was quickly followed by liquid nitrogen cryopreservation and storage. Homogenates were made by homogenization and centrifugation at 2500 r/min and 4 °C for 20 min. The supernatant were carefully collected. To acquire the plasma samples, the blood was centrifuged at 5000 rpm for 6 min. The plasma cytokines and hippocampal PAF were measured by highly sensitive ELISA kits (CUSABIO, China), following the manufacturer’s instructions.

### Immunohistochemistry and Nissl staining

After depication under pentobarbital solution (50 mg/kg, intraperitoneally), the brain tissues were rapidly fixed in paraformaldehyde and embedded in paraffin. The tissues were cut into 4-μm sections. The sections were dewaxed and antigen repaired according to commonly used protocols, and the sections were incubated in 0.3% hydrogen peroxide at room temperature for 10 min to inactivate the endogenous enzymes. BSA (5%) was used to block the nonspecific binding sites. The hippocampal tissue sections were incubated with the specific primary antibody, anti-Iba1 antibody (1:1000, Abcam, 178,846). Then the tissue sections were incubated with the biotinylated secondary antibody. We used diaminobenzidine to detect the signals and used hematoxylin to stain the cell nuclei. For Nissl staining, the sections were dewaxed and washed with distilled water. Then, the sections were dyed in a cresyl violet stain solution at 37℃ for 1 h. The sections were rapidly differentiated in 95% ethanol, and the purple Nissl bodies were observed by microscopy. Then, we dehydrated the sections with gradient alcohol, cleared the sections with xylene, dried and covered the sections with glass before microscopy. For quantification, the activated microglia or surviving neurons in five randomly selected fields of the hippocampal CA3 area were calculated based on the mean integrated optical density, which was counted at 200 × magnification. An Olympus microscope and ImageJ software were used for image analysis.

### Western blotting analysis

The total hippocampal tissue proteins were extracted by homogenization with RIPA lysis buffer supplemented with 1 mM protease inhibitor cocktail and 1 mM phenyl methylsulfonyl fluoride (PMSF). The tissue lysates were purified by centrifugation for 15 min at 4 °C. The protein concentration was measured by a BCA Protein Assay Kit (Thermo Fisher). Equal amounts of each tissue protein sample were boiled with 5 × sample loading buffer for 5 min at 95 °C. The proteins (80 μg) were separated by 12% SDS-PAGE and transferred onto PVDF membranes. The membranes were blocked with 5% skim milk for 1 h at room temperature and were incubated with primary antibodies against Iba-1 (1:1000, Abcam, 178,846) at 4 °C overnight. After washing 3 times with TBST, the secondary HRP-labeled goat anti-rabbit IgG antibody was used to label the primary antibodies. After washing 3 times, the protein bands were detected with a chemiluminescent HRP substrate (Millipore) on a Bio-Rad ChemiDoc XRS system.

### Statistical analysis

An observer who was blinded to the experiment analyzed all the data. The results are expressed as the mean ± standard error of the mean (SEM). Comparisons between two groups were performed with t-test. Data among 3 groups were analyzed with one-way analysis of variance (ANOVA), followed by Bonferroni’s test for post hoc multiple comparisons. In all cases, a *p* value less than 0.05 was considered statistically significant.

## Results

### Enhancement of PAF in the partial hepatectomy group

**Fig.1:**
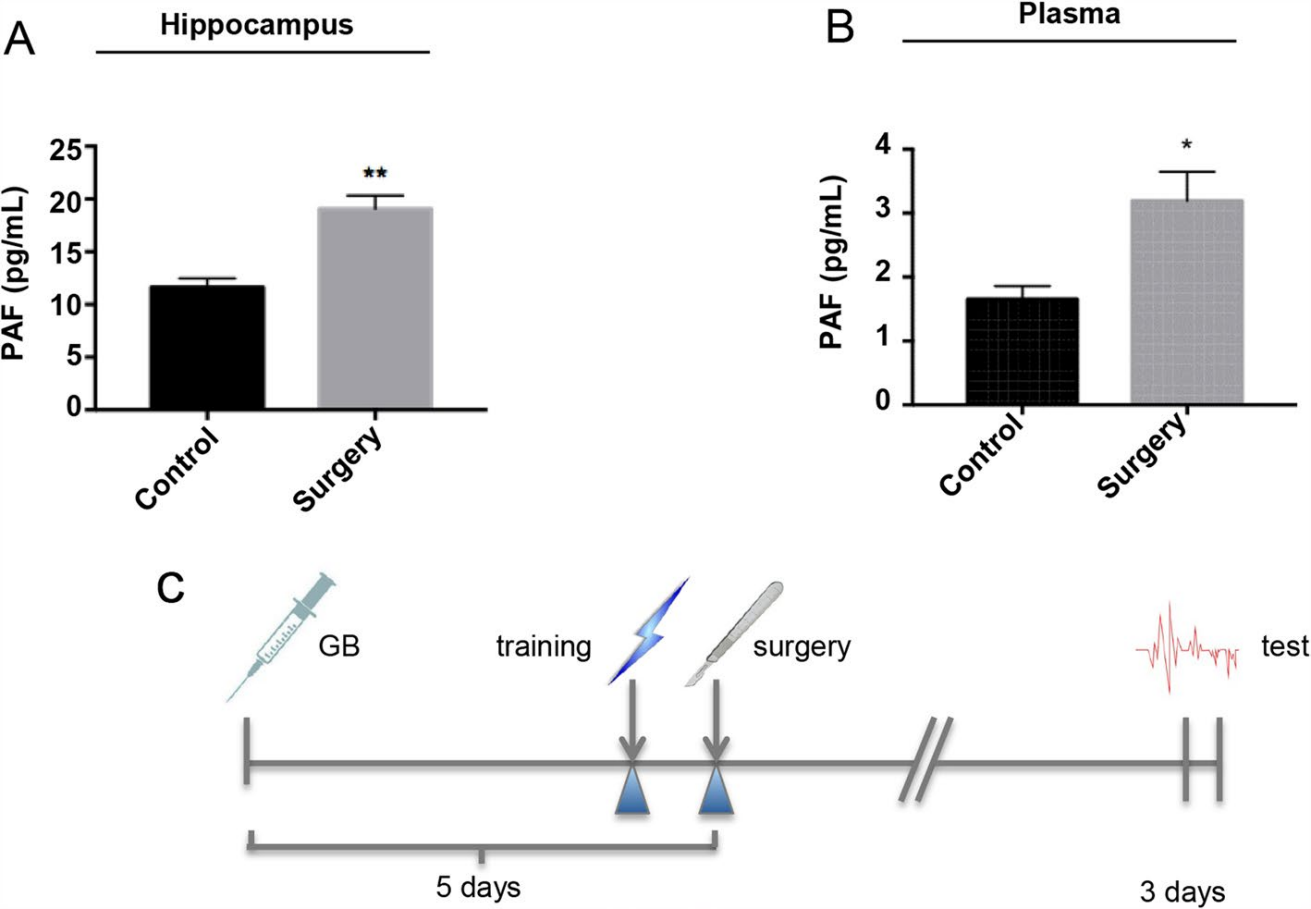
Expression of PAF in the hippocampus and plasma. The concentration of PAF was measured by ELISA on the 3rd day after surgery. **A** The surgery group showed elevation of PAF level in the hippocampus (*n* = 6 per group) and **B** plasma (*n* = 10 per group). **C** Timeline of the experiment. (Data are expressed as the means ± SEMs; *p < 0.05, **p < 0.01 vs. control group)

To determine the involvement of PAF in the progression of POCD, we measured the PAF level by ELISA. The PAF content in the plasma and hippocampal homogenates was increased in the surgery group compared to the control group (*p* < 0.05) (Fig. 1A, B). Compared with the control group, the surgery group exhibited enhanced levels of PAF in the plasma and hippocampus by 1.93-fold and 1.54-fold, respectively. These results indicated that PAF might be associated with the POCD model established with partial hepatectomy surgery.

### GB improved POCD induced by partial hepatectomy in aged rats

**Fig.2:**
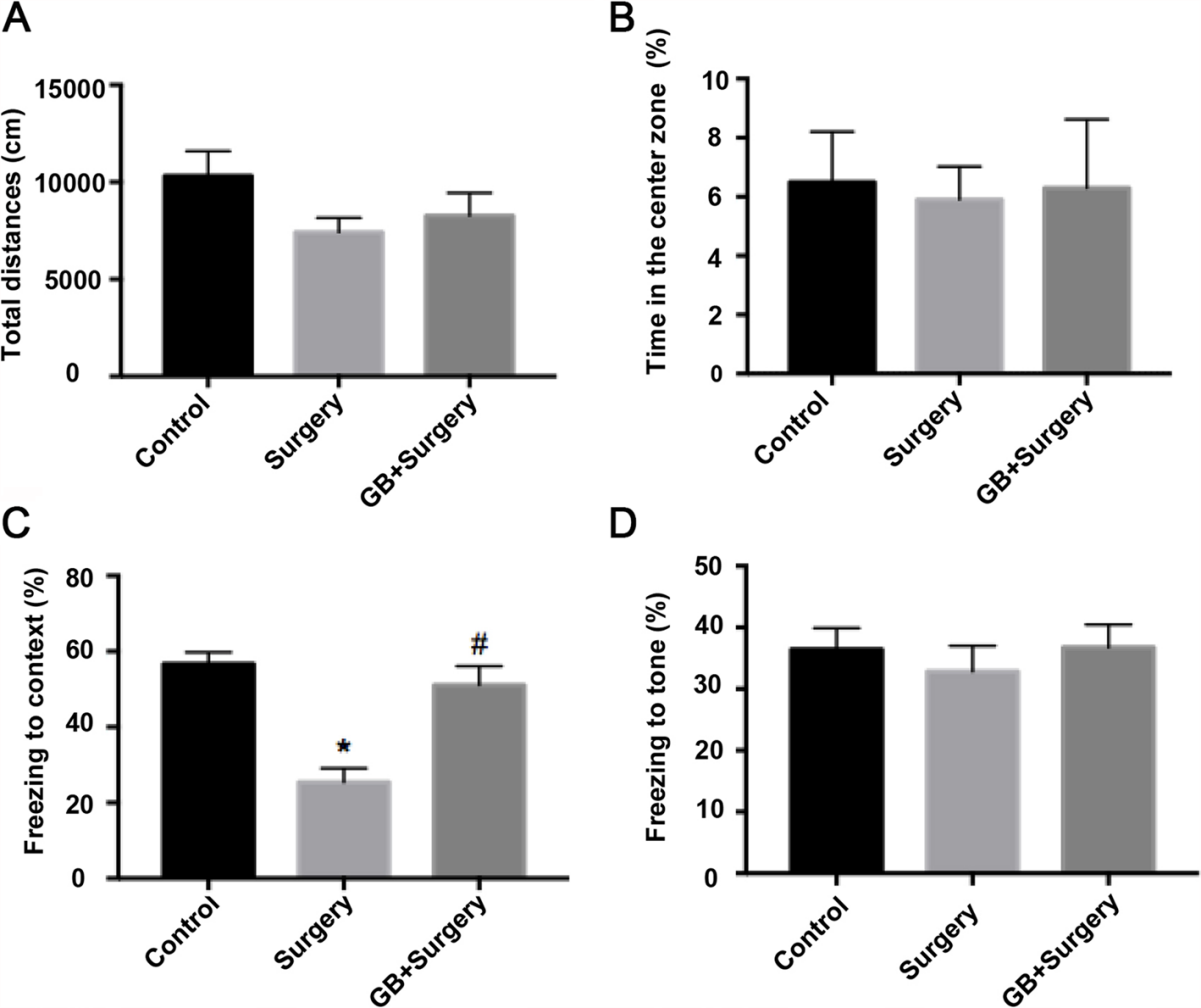
Results of neurobehavioral tests. **A** There was no significant difference in total distance (cm) and **B** time spent in the center of the open field among the three groups. **C** In the fear conditioning test, the freezing time to context was significantly decreased in the surgery group compared with the control group, while pretreatment with GB increased the freezing time. **D** There was no significant difference in the cued fear conditioning test among the groups. (*n* = 10 per group) (Data are expressed as the means ± SEMs; *p < 0.05 vs. control group ^#^*p* < 0.05 vs. surgery group.)

To determine the effects of GB on surgery-induced cognitive impairment, aged rats were intraperitoneally injected with GB for 5 days before the partial hepatectomy surgery. The open field test was used to evaluate exploratory activity and anxiety-like behavior, while the trace fear conditioning test was used to assess cognitive ability after surgery (Fig. 1C). In the open field test, the total distance traveled and the time spent in the center zone were not different among the three groups (Fig. 2A, B, *p* > 0.05). In the contextual fear conditioning test, the percentage of freezing time in the operation group decreased significantly compared with that in the control group, and this decrease was prevented by the preoperative administration of GB (Fig. 2C, *p* < 0.05). In the cued fear conditioning test, the freezing time was not significantly different among the three groups (Fig. 2D, *p* > 0.05). These results indicated that hippocampus-dependent memory function decreased after partial hepatectomy, and GB was able to reduce the cognitive impairment induced by surgery.

### GB inhibited the excessive microglial activation induced by surgery

**Fig. 3:**
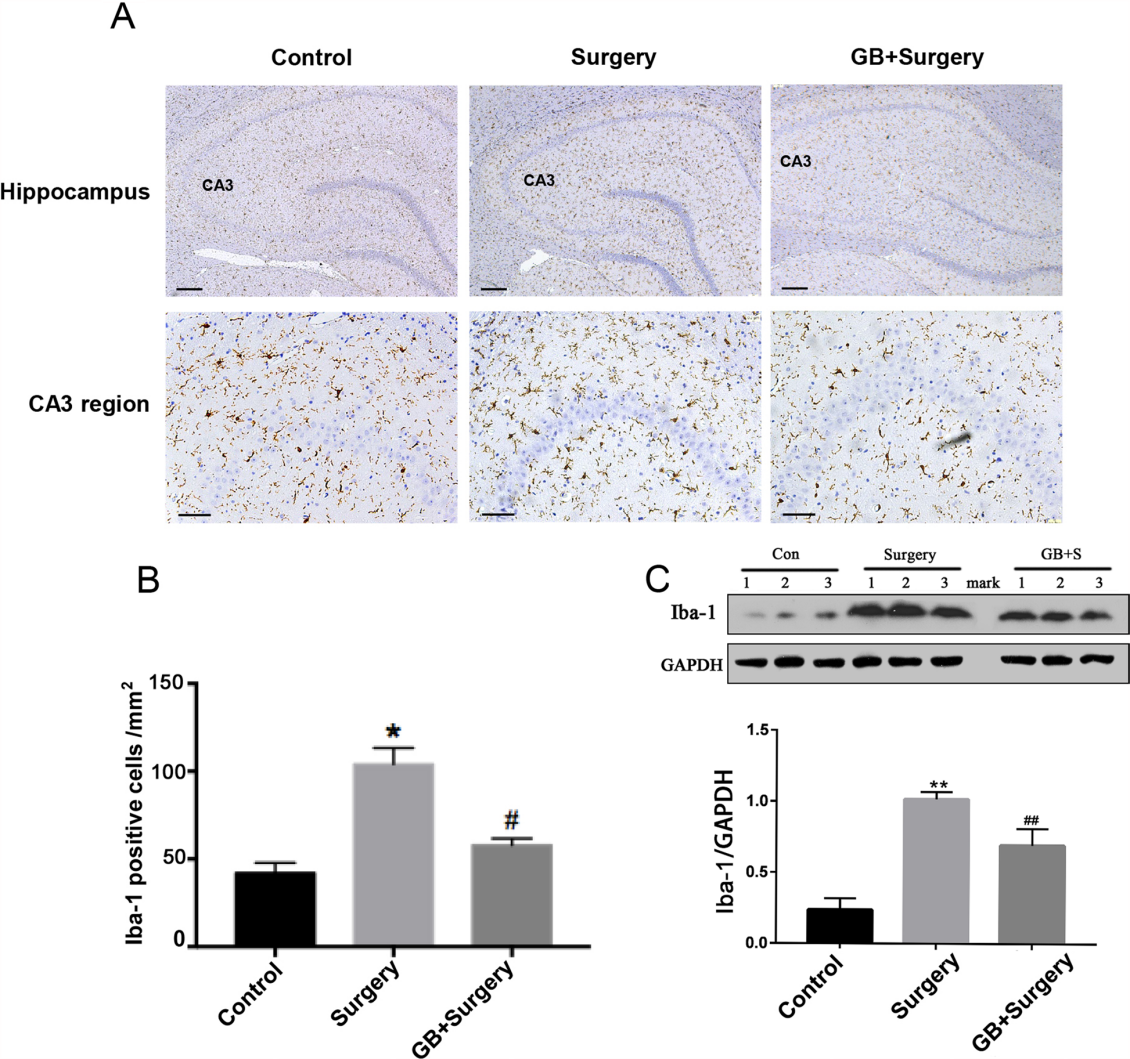
Pretreatment with GB significantly inhibited surgery-induced microglial activation. **A** Representative images of immunostaining of Iba-1, **B** The number of microglia was remarkably increased in the surgery group compared with the control group. GB significantly inhibited microglial activation in the hippocampus induced by the partial hepatectomy surgery. **C** Overexpression of Iba-1 could be decreased by pretreatment with GB. 500um in top row and 100um in bottom row of A. (*n* = 4 per group). (Data are expressed as the means ± SEM; *p < 0.05, **p < 0.01 vs. control group; ^#^*p* < 0.05, ^##^*p* < 0.01 vs. surgery group)

Immunohistochemical staining and western blotting were conducted to assess the expression of the ionized calcium binding adaptor molecule-1 (Iba-1) protein, which is a marker of microglia. The immunohistochemistry results showed that partial hepatectomy surgery under isoflurane anesthesia caused obvious microglial activation in the rat hippocampal CA3 regions, which was inhibited significantly by pretreatment with GB (*p* < 0.05) (Fig. 3A, B). In accordance with these results, western blot analysis showed increased expression of Iba-1 in the hippocampus of the operation group, which was inhibited by treatment with GB (*p* < 0.05) (Fig. 3C).

### GB inhibited the excessive microglial activation and increased proinflammatory cytokines induced by surgery

**Fig.4:**
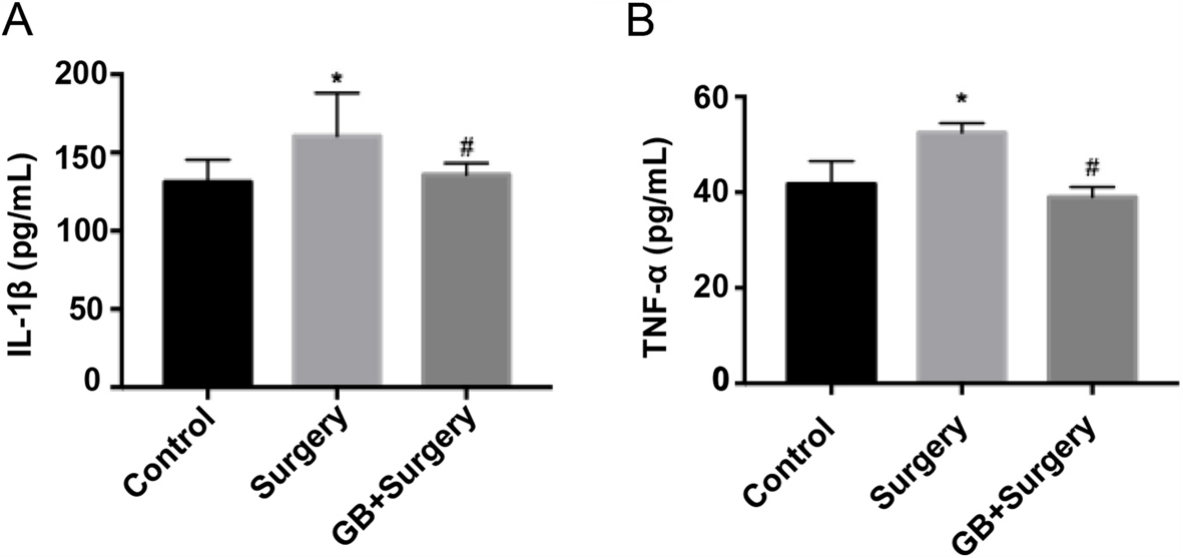
Effects of GB on proinflammatory cytokines in the hippocampus. (**A**)(**B**) The rats in the surgery group showed an elevation of IL-1β and TNF-a level in hippocampus. While pretreatment with GB inhibited the elevation of IL-1β and TNF-a induced by surgery. (*n* = 6 per group) (Data are expressed as the means ± SEM; *p < 0.05 vs. control group;.^#^*p* < 0.05, vs. surgery group)

The expression of the proinflammatory cytokines TNF-α and IL-1β in the hippocampus of surgery group was significantly increased compared to that in the control group. While the expression levels of TNF-a and IL-1β in the GB group had no significant difference with the control group (Fig. 4*p* < 0.05).

### Neuroprotective effect of GB on POCD

**Fig. 5:**
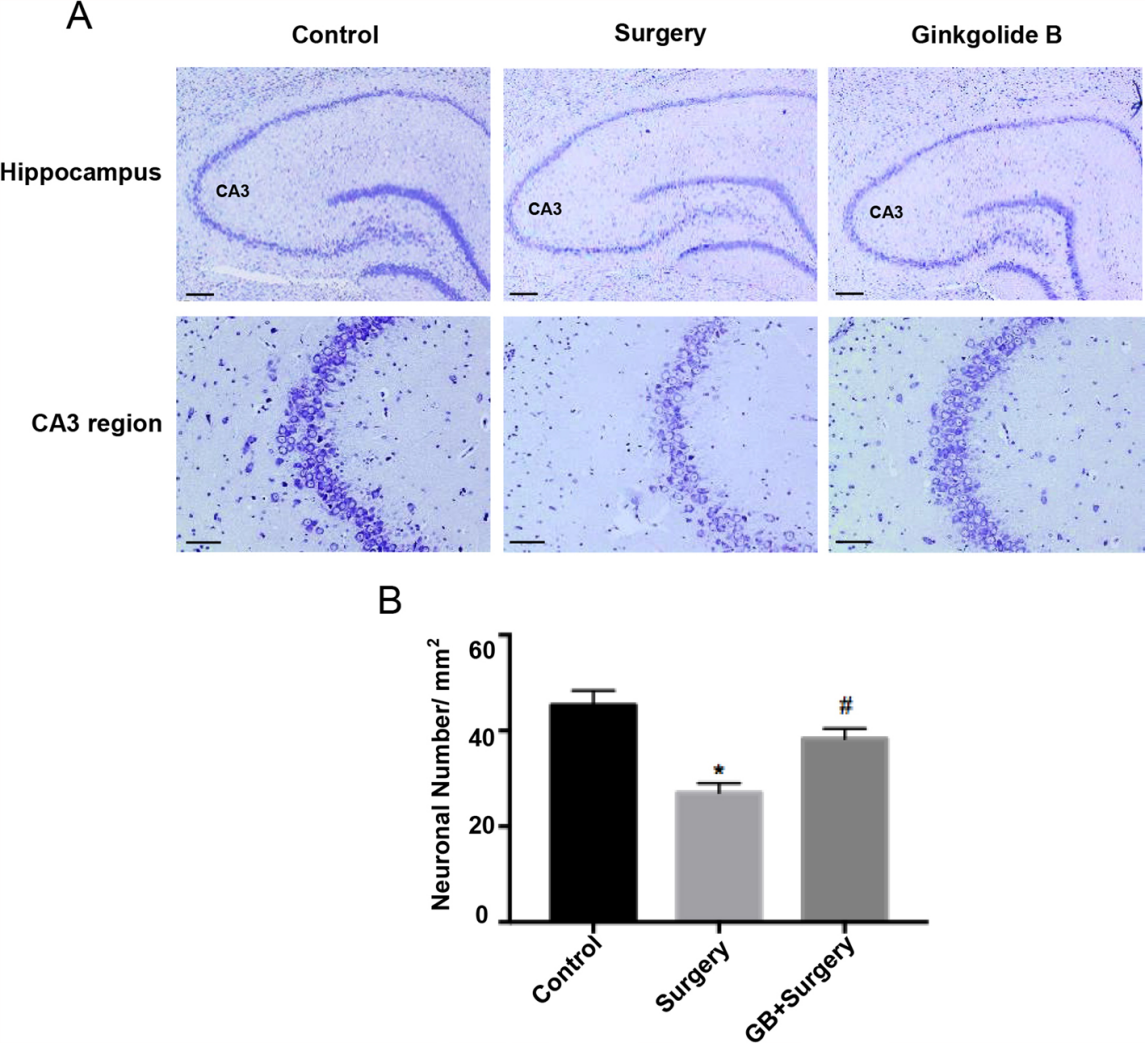
Neuroprotective effect of GB against POCD in aged rats. (**A**)(**B**) Nissl staining showed a reduced number of neurons in the surgery group compared to the control group, while pretreatment with GB prevented the surgery-induced neuronal loss. Scale bars: 500um in the top row and 100um in the bottom row of A. (*n* = 4 per group). (Data are expressed as the means ± SEMs; *p < 0.05, **p < 0.01 vs. control group; ^#^*p* < 0.05 vs. surgery group.)

To confirm the protective role of GB in POCD, we examined the effect of GB on the overall number of neurons after surgery and anesthesia by Nissl staining. The number of neurons in the hippocampal CA3 area of the surgery group was reduced compared to that of the control group. However, GB pretreatment notably reduced the surgery-induced neuronal loss (*p* < 0.05) (Fig. 5A, B). Together, these data indicated that GB is neuroprotective after surgery.

## Discussion

This paper showed the enhancement of PAF in the hippocampus and plasma of rats with POCD. The main finding of this study was that, the administration of GB, an antagonist of PAFR, prevented POCD by suppressing microglial activation and subsequently reducing inflammatory mediator release. Our results suggested that GB may be an effective treatment for POCD.

Elderly is at high risk of POCD, therefore we used 21 ± 1 month-old rats in the present experiment, and these rats can be considered as aged rats. We established a model of POCD by partial hepatectomy in accordance with a previous study [23]. The cognitive function after surgery was assessed by open field test and trace fear conditioning test, which are well-validated assays of learning and memory and often used in the laboratory animal [25]. Since previous studies showed cognitive decline and elevated inflammation on 3rd day after surgery, we performed behavior tests and collected sample at this point in this study [26]. Behavioral tests showed cognitive dysfunction in aged rats after surgery.

PAF was involved in the impairment of synaptic plasticity and in deficits in learning and memory deficit [27]. Long-term potentiation (LTP) has been considered as the basis of learning and memory. PAF was shown to attenuate LTP in hippocampal slices [20]. We demonstrated increased PAF in the hippocampus and plasma of rats with POCD for the first time. This observation suggested an association between increased PAF expression and POCD. PAF has been recognized as an important inflammation mediator. The stimulation of PAFR can regulate the expression of intracellular genes by promoting the FOS/ JUN/AP-1 transcriptional signaling pathway and the transcription of COX-2, which correlate with microglial functions [28, 29].

Accumulating evidence has demonstrated that activation of microglia plays an important role in POCD by promoting neuroinflammation, as evidenced by the increased release of proinflammatory cytokine, such as TNF-a, IL-1β and IL-6 [7]. The depletion of microglia can protect mice from POCD, even mice with high risk factors for POCD, such as lipid metabolism disorder, and aging [30]. Chemicals, such as berberine, which can inhibit the activation of microglia, could alleviate POCD too. [31]. It’s well-known that hippocampus serves a critical role in learning and memory [32]. Hence, we tested the microglial activation and proinflammatory cytokine in hippocampus after surgery, as previous studies do [33]. Confirming the finding of previous studies, we found activated microglia and increased IL-1β and TNF-a in the hippocampus on the 3rd day after surgery.

Nissl staining is a commonly used technique to determine the injury of neurons [34]. We employed this technique to examine the surviving neurons in hippocampus on the 3^rd^ day after surgery. As previous studies do, the CA3 regions of the hippocampus was selected to demonstrate the Nissl staining results [21]. Neuronal loss was markedly increased in the surgery group compared with the control group.

GB is a natural and strongest antagonist of platelet-activating factor receptor. A previous study showed that blockade of PAF receptor attenuates abnormal behaviors induced by phencyclidine through the downregulation of NF- κB, which is involved in inflammation [35]. GB alleviates the inflammation in rat brain tissues caused by ischemia–reperfusion, beta-amyloid toxicity and intracerebral hemorrhage [15, 36, 37]. GB is able to pass through blood–brain barrier after systemic administration [36]. Considering the fact that GB has been used in the clinic, we speculate that GB would be therapeutically effective for POCD. Based on the dose of GB in previous studies [36], we performed pretest study with GB at different dose (5 mg/kg, 10 mg/kg, 15 mg/kg, ip, 5 days), and we found the dose at 10 mg/kg was able to prevent PND. Prelimilary study results were not shown here. Consistently, our study demonstrated that pretreatment of GB inhibited the microglial activation induced by surgery and reduced the levels of proinflammatory cytokines (IL-1β and TNF-α) in the hippocampus and plasma. In addition, we found that GB could maintain the integrity of neurons, and alleviate the decline of cognitive function induced by surgery.

There are several limitations to our study. First, using PAF siRNA would provide more specific evidence regarding whether the depletion of PAF could improve learning and memory abilities after surgery. As the next step, we will perform experiments with PAF siRNA to explore new pathways involved in POCD. Second, analyzing the activation of microglia and levels of proinflammatory factors at different time points would improve our understanding of the development of POCD. To minimize the animals used, we observed the abovementioned indicators on the 3rd day after surgery, in accordance with previous findings [38]. Moreover, there may be additional mechanisms through which GB prevents POCD. Therefore, advanced experiments are urgently needed to provide a deeper understanding of the neuroprotective effects of GB against POCD.

Overall, GB, which is a platelet-activating factor receptor antagonist, can protect against the progression of POCD. The molecular and specific mechanisms still need to be studied. These studies are expected to be enlightening to subsequent research. 

## Conclusions

Our findings suggested that PAF was involved in the development of POCD. Improvement of POCD by GB was associated with the inhibition of microgliosis-mediated neuroinflammation and neuronal apoptosis in aged rats.

## Supplementary Information

**Additional file 1.**

## Data Availability

All data presented in this study are available from corresponding author on reasonable request.

## Abbreviations

ANOVA: One-way analysis of variance
BSA: Bovine serum albumin
ELISA: Enzyme-linked immunosorbent assay
IBA-1: Ionized calcium-binding adapter molecule-1
IL-1β: Interleukin-1β
IL-6: Interleukin-6
PAF: Platelet-activating factor
TFC: Trace fear conditioning
TNF-α: Tumor necrosis factor-α
