# Supplementary figures and images for: Ginkgolide B improved postoperative cognitive dysfunction by inhibiting microgliosis-mediated neuroinflammation in the hippocampus of mice
Source: BMC Anesthesiol. 2022 Jul 18;22:229. doi: 10.1186/s12871-022-01750-1 (PMC9290278; doi:10.1186/s12871-022-01750-1)

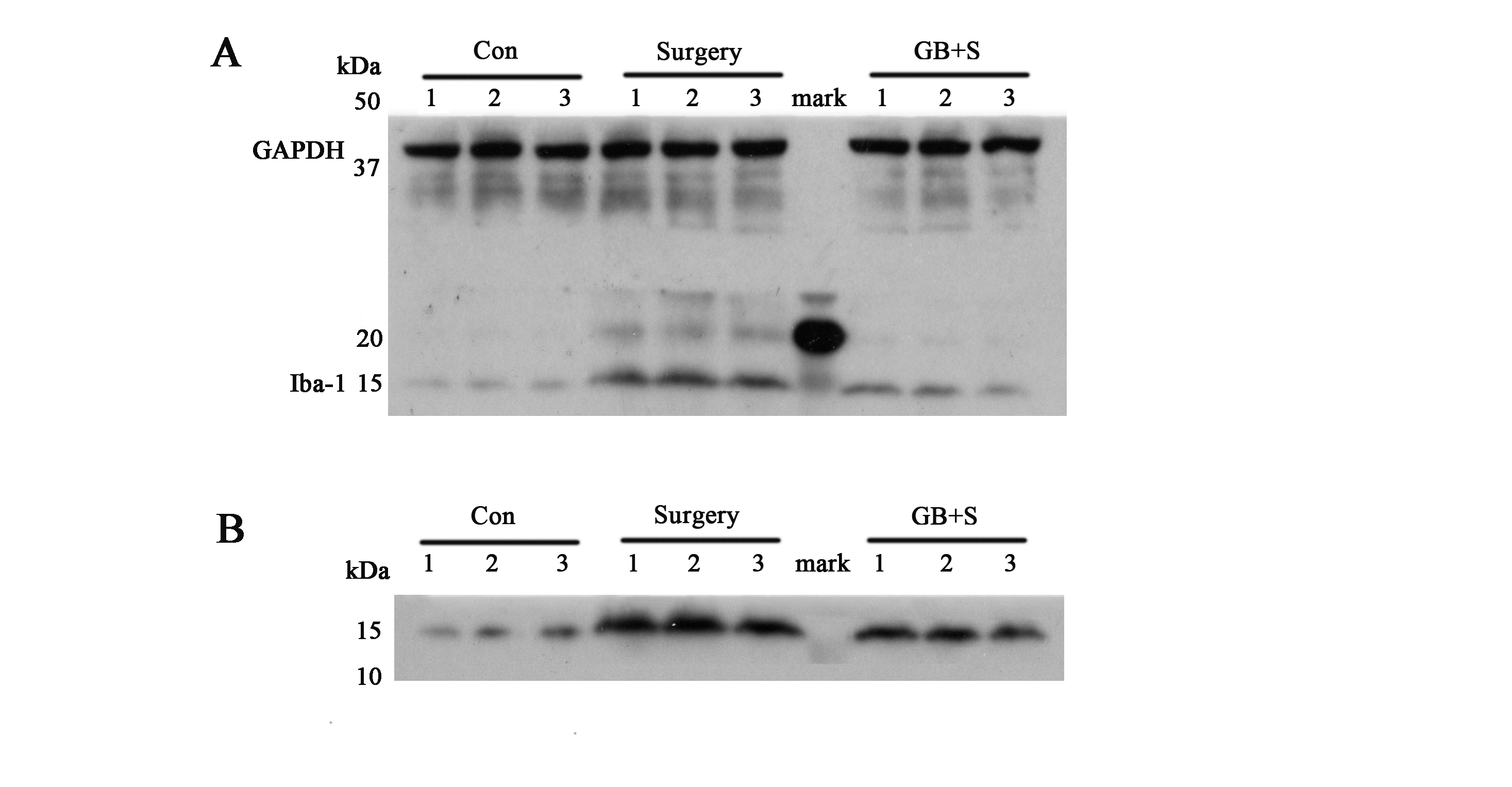

Supplement: Supplementary file 1 — Additional file 1. [file 12871_2022_1750_MOESM1_ESM.tif]
